# One year pre-diagnostic prevalence and associated factors of polypharmacy and potentially inappropriate medication use in community-dwelling adults with mild cognitive impairment or dementia in Norway (2014–2024): A registered report protocol

**DOI:** 10.1371/journal.pone.0356275

**Published:** 2026-08-18

**Authors:** Hege Kersten, Tonje Marie Bø Vaksvik, Rita Romskaug, Torgeir Bruun Wyller, Keson Jaioun, Edoardo Botteri, Geir Selbæk

**Affiliations:** 1 Department of Research, Telemark Hospital Trust, Skien, Norway; 2 Institute of Clinical Medicine, Faculty of Medicine, University of Oslo, Oslo, Norway; 3 Norwegian National Centre for Ageing and Health, Vestfold Hospital Trust, Tønsberg, Norway; 4 Department of Geriatric Medicine, Oslo University Hospital, Oslo, Norway; 5 Department of Colorectal Screening, Cancer Registry of Norway, Norwegian Institute of Public Health, Oslo, Norway; 6 Department of Research, Cancer Registry of Norway, Norwegian Institute of Public Health, Oslo, Norway; Qatar University, QATAR

## Abstract

**Background:**

People with cognitive impairment frequently have multiple comorbidities, and polypharmacy is highly prevalent, affecting nearly half of individuals with mild cognitive impairment and dementia. The use of potentially inappropriate medications (PIMs), including those specifically problematic for cognitive impairment (PIMcog), increases with the total number of medications and may exacerbate cognitive symptoms or increase the risk of adverse drug events. However, the prevalence of polypharmacy and PIMcog use among patients diagnosed with MCI or dementia in Norwegian specialist outpatient clinics has not been described.

**Methods:**

We will conduct a retrospective cohort study linking national health registries to estimate the prevalence of polypharmacy and PIMcog use in the year prior to diagnosis of mild cognitive impairment or dementia. Data from the Norwegian Registry of Persons Assessed for Cognitive Symptoms will be linked to dispensing data from the Norwegian Prescribed Drug Registry and comorbidity data from the Norwegian Patient Registry. The study population will include all patients diagnosed with mild cognitive impairment or dementia in Norwegian specialist outpatient clinics from 2014 to 2024. We will estimate the twelve-month prevalence of polypharmacy and PIMcog use preceding diagnosis, compare sociodemographic and clinical characteristics between PIMcog users and non-users, and identify factors associated with PIMcog use.

**Expected impact:**

By describing the one-year prevalence and patterns of polypharmacy and PIMcog use and associated factors prior to mild cognitive impairment and dementia diagnosis, the findings may inform targeted deprescribing interventions and safer prescribing strategies for individuals with cognitive impairment.

## Introduction

The risk of developing dementia and mild cognitive impairment (MCI) increases markedly with age. By 2050, one-fifth of Norway’s population of 6.2 million will be aged ≥70 years [1]. In 2025, the prevalence of dementia in Norway is estimated at 115,000, and this number is expected to more than double to 237,000 by 2050 [2]. As the prevalence of dementia rises, there is increasingly focus on identifying modifiable risk factors that may be targeted to delay disease onset and mitigate the symptom burden [3].

The Norwegian Registry of Persons Assessed for Cognitive Symptoms (NorCog) was established as a national quality registry in 2013. The purpose of NorCog is to harmonize and improve the diagnostic work-up across specialized healthcare units assessing persons with cognitive symptoms [4]. In 2024, there were 46 clinics providing data to the registry. In total, 27,500 patients are included in the register, with a mean age of about 74 years. Almost 85% of these patients have been diagnosed with MCI or dementia. NorCog constitutes a large research cohort with extensive diagnostic and treatment data and has contributed to 74 published studies [5].

Multiple comorbid conditions develop with aging, and the prevalence of comorbid medical illnesses in individuals with dementia increases with dementia severity [6]. Older adults with dementia have more than twice the burden of physical comorbid conditions compared to age- and sex-matched controls without dementia [7]. Common coexisting conditions in dementia include depression, diabetes, coronary heart disease, stroke, hypertension, heart failure, and incontinence [7–9]. These conditions are typically managed with multiple medications. In addition, pharmacological treatment is often used to manage cognitive symptoms and behavioral and psychological symptoms of dementia (BPSD) [10,11]. Consequently, around 50% of individuals with dementia are exposed to polypharmacy, and their average medication use is higher than that of individuals without dementia [6,12]. In a population-based study from Norway, including 187 community-dwelling patients with Alzheimer’s dementia and 200 controls, the age- and sex-adjusted mean number of medications was 5.1 ± 3.6 among individuals with Alzheimer’s dementia compared to 2.9 ± 2.4 among cognitively healthy controls [13].

According to the Screening Tool of Older Persons’ Prescriptions (STOPP) and the Beers criteria, widely used prescription tools in geriatric medicine, CNS-depressants such as benzodiazepines (BZDs), BZD-related drugs, certain antipsychotics, and drugs with strong anticholinergic effects are considered potentially inappropriate medications for individuals with cognitive disorders (PIMcog) [14,15]. This inappropriateness is due to drug–disease interactions, which can exacerbate cognitive decline and functional impairment [16–19]. Despite their risks, PIMcog are still commonly prescribed for managing BPSD. In the United States, 82% of patients referred to a hospital for evaluation of cognitive decline used sedatives or anticholinergic drugs [18]. Other studies have shown a 20–30% prevalence of PIMcog in community-dwelling older adults with cognitive disorders [16,20]. A systematic review, including 11 studies from memory clinics in eight different countries, found PIM- and polypharmacy rates of 38% and 60%, respectively, with significant variation between countries. No prevalence studies from the Nordic countries were included in the review [21]. Prescription patterns and PIMcog prevalence vary with local pharmacotherapeutic practices, highlighting the need for studies in Nordic specialist outpatient clinics. No such prevalence studies have previously been conducted in Norway, but trends of psychotropic drug use in nursing homes and among community-dwelling individuals with dementia have shown a decrease in antipsychotic drug use in the last decades, but only minor changes for the other psychotropic drugs [22,23].

The Norwegian Prescribed Drug Registry (NorPD) contains detailed information on all prescriptions dispensed at Norwegian pharmacies, recorded at the individual level using unique national personal identifying number. This enables linkage to other national health registries [24]. In this registry based study, we link NorCog data with NorPD to estimate the twelve-month prevalence of polypharmacy and PIMcog use prior to diagnosis of MCI or dementia in Norwegian outpatient clinics. In addition, we will incorporate information on comorbidities to identify clinical characteristics associated with PIMcog use. The findings may inform targeted screening and deprescribing strategies, improve medication safety, and support optimization of pharmacological treatment in this vulnerable population [21].

### The present study

This study aims to estimate the 12-month prevalence of polypharmacy and potentially inappropriate cognitive-impairing medications in the year preceding a diagnosis of MCI and dementia in Norwegian specialist outpatient clinics. By characterizing prescribing patterns and identifying patient characteristics associated with PIMcog use, the study seeks to inform targeted deprescribing strategies and improve the quality of pharmacological care. Ultimately, the goal is to reduce drug-related cognitive decline and preserve functional autonomy and quality of life in individuals living with MCI and dementia.

### Objectives and hypotheses

The objectives of the present study are to:

1] Estimate the 12-month prevalence of polypharmacy (≥5 concurrent medications) and PIMcog - including benzodiazepines, benzodiazepine derivatives, benzodiazepine-related drugs, anticholinergics and antipsychotics- among community-dwelling individuals in the year prior to a diagnosis of MCI or dementia in Norwegian outpatient clinics during the period 2014–2024.
2] Compare the characteristics of PIMcog users and non-users, and identify factors associated with PIMcog use among community-dwelling individuals diagnosed with MCI or dementia who are registered in NorCog from 2014 to 2024.

We hypothesize that:

1) The prevalence of polypharmacy has increased over the study period, while PIMcog prescribing patterns have changed and vary across regions.
2) PIMcog use is associated with greater disease burden, including more severe dementia, higher comorbidity, older age, higher overall medication use, and a higher prevalence of BPSD.

## Materials and methods

### Design and setting

This is a registry based retrospective cohort study linking data from NorCog, NorPD, and the Norwegian Patient Registry (NPR).

The primary outcomes of the study are the 12-month prevalence of polypharmacy and PIMcog use among community-dwelling individuals diagnosed with MCI or dementia in Norwegian specialist outpatient clinics during the study period from 2014 to 2024.

In addition, the study will identify factors associated with PIMcog use and characterize temporal trends and regional variation in prescribing patterns over the study period.

### Data collection

#### Selection of the study population registered in NorCog.

NorCog collects data according to a standardized assessment manual in hospital outpatient clinics in Norway, with the aim of harmonizing and improving the diagnostic work-up for individuals referred for assessment of cognitive symptoms and suspected dementia [4]. Patients are encouraged to bring a family member or another close informant with regular contact and sufficient knowledge of the patient’s cognitive and functional status. NorCog was established in 2008 with seven participating outpatient clinics, expanding to 46 outpatient clinics by the end of 2024. NorCog became a national medical quality registry in 2013 and reported a coverage ratio of 70% for individuals diagnosed with dementia in specialized healthcare units in Norway in 2024 [5].

Participation in NorCog was voluntary during the study period and required informed consent. Until the end of 2021, only patients with the capacity to provide written informed consent were eligible for inclusion. Since January 2022, patients lacking capacity to provide written informed consent could be included based on proxy consent.

According to the *American Geriatrics Society Beers Criteria®* and STOPP version 3, both prescribing tools intended for individuals aged ≥65 years, PIMcog should be avoided in those with cognitive impairment. In this study, we will identify PIMcog among community-dwelling patients referred to specialist outpatient clinics who are diagnosed with MCI or dementia and registered in NorCog 01/01/2014 to 12/31/2024. As NorPD does not record information on medications dispensed in institutional care, only community-dwelling patients are eligible for inclusion in this study.

Diagnoses are assigned following comprehensive clinical assessments in outpatient clinics specialized in the assessment and diagnosis of cognitive impairment and dementia. The assessments include medical history, cognitive testing, evaluation of functional status, clinical examination, and information from informants. When relevant, neuroimaging and laboratory investigations are also performed. Diagnostic classification is based on established criteria, with MCI defined according to the Winblad criteria and dementia diagnosed according to ICD-10 [25,26]. Where possible, further etiological subtyping is performed using recognized research criteria, including the National Institute on Ageing–Alzheimer’s Association workgroups on diagnostic guidelines for Alzheimer’s disease criteria for Alzheimer’s disease, VASCOG criteria for vascular dementia, McKeith criteria for dementia with Lewy bodies, Emre criteria for Parkinson’s disease dementia, and Rascovsky and Gorno-Tempini criteria for frontotemporal dementia variants [27–32].

**Fig 1:**
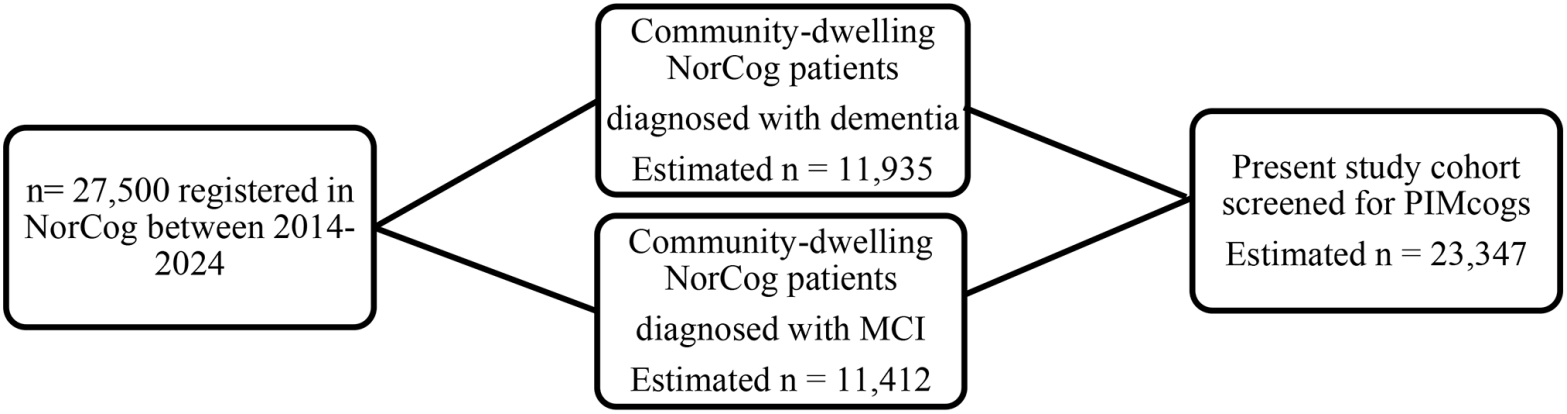
Extraction of the study cohort from NorCog.

In total, 27,500 patients from 46 outpatient clinics from all four regional health authorities in Norway have been registered in NorCog since 2014 [5]. In 2024, 43.4% were diagnosed with dementia, and 41.5% with MCI, while the rest had subjective cognitive impairment, no cognitive impairment or other specified diagnosis such as mood disorders [5]. Based on the 2024 numbers, we expect that approximately 85% of the NorCog patients from 2014–2024 will be eligible for inclusion. Selection of the study cohort is shown in Fig 1.

#### Extraction of variables from NorCog (Table 1).

Demographic, social and clinical variables potentially associated with PIMcog use will be extracted from NorCog and compared between PIMcog users and non-users of PIMcog. The selected demographic variables allow us to analyze differences related to local pharmacotherapeutic traditions and prescription patterns. The social variables are chosen due to possible differences in drug use related to age group, sex, living conditions, and the presence of family carers [37].

**Table 1:** Selected variables extracted from NorCog.

Explanatory variables	Measures/Questionnaire	Assessing/Coding/Scoring
Demographic	Health region, hospital, postal code, date of first visit	
Social (I,P)	Age Sex Education Occupational activity today Marital status Residential care Living alone Receiving health care services Time since symptom onset	Years Female/Male (<9 years, 9–12 years, > 12 years) Yes/No (Single/Maried or cohabiting/Widowed/Divorced) Yes/No* Yes/No Yes/No Months
Somatic health (D)	Norwegian version of Clinical Frailty Scale (NR-CFS)** [33] Present or previous physical and/or psychiatric disease	Sum score 1–9, higher score indicates greater frailty, 1 is very fit and 9 is terminally ill Cerebrovascular, other CNS disorders, cancer, arthritis, cardiovascular, endocrine, chronic obstructive pulmonary disease, psychiatric disease
Neuropsychiatric symptoms (I)	Neuropsychiatric Inventory Questionnaire (NPI-Q) [34] Norwegian version of Cornell Scale for Depression in Dementia (CSDD) [35]	Assessing the presence and severity (0–3) of psychopathological symptoms within 12 areas, maximum score is 36 Sum score 0–38, higher score indicating more depressive symptoms, and score ≥ 6 indicates depressive episode in memory clinic patients [9]
Diagnosis (D)	MCI (F06.7/F07.8) [26] Dementia (ICD-10)	Measured cognitive impairment on at at least one cognitive test (cut-off 1.5 SD below age- and education-adjusted norms) and/or measured cognitive decline, no significant reduction in ADL function Impairment in two or more cognitive domains with impairment in activities of daily living, preserved consciousness, and decline in emotional control, motivation, or change in social behavior
Dementia etiology (D) (ICD-10)	Early-onset Alzheimer's dementia Late-onset Alzheimer's dementia Alzheimer's dementia, atypical or mixed type Vascular Dementia Frontotemporal Dementia Dementia with Lewy bodies Parkinson's disease dementia Unspecified dementia Other dementia	F00.0 and G30.0 F00.1 and G30.1 F00.2 and G30.8 F01.X F02.0 and G31.0 F02.8 and G31.8 F02.3 and G20 F03
Dementia severity (D)	Clinical Dementia Rating (CDR) [36]	Rates cognition and functional performance (0 = no dementia, 0.5 = MCI, 1 = mild dementia, 2 = moderate dementia, 3 = severe dementia) within six domains: memory, orientation, judgment and problem-solving, community affairs, home and hobbies, and personal care. Maximum score is 18

*People in residental care will be excluded from further analyses. **CFS is only available from individuals registered in NorCog from 2016

(P) Source of information is the patient, sometimes supplied by the informant. (I) Source of information is the informant

(D) Source of information is the Doctor specialized in diagnosing dementia

Somatic illnesses and comorbidity burden have been shown to be associated with polypharmacy and the risk of inappropriate drug use [6,38]. Measure of somatic health and comorbidity will include recorded diagnoses and the Clinical Frailty Scale (CFS) score from NorCog [33]. In addition, the Charlson Comorbidity Index (CCI) will be derived from diagnoses registered in the NPR [39].

NPR is a national health registry that contains individual-level data for all patients who receive treatment, or are awaiting, the specialist health care services in Norway [40]. BPSD will be assessed using scores from the Neuropsychiatric Inventory Questionnaire (NPI-Q) and the Cornell Scale for Depression in Dementia (CSDD) [34,35,41]. Dementia etiology and severity will also be extracted from NorCog.

#### Drug prescriptions extracted from the Norwegian Prescribed Drug Registry (NorPD).

The NorPD consists of information about all prescription drugs dispensed from pharmacies to individuals but does not include medications dispensed in institutions. Therefore, only community-dwelling patients are included in this study. All information about drug use in the study cohort will be extracted from the NorPD, including date of dispensing, medicinal product name and formulation, complete Anatomical Therapeutic Chemical (ATC) code, and the quantity of dispensed medications.

Drug prescriptions dispensed to the study participants from pharmacies during the twelve months preceding the diagnostic assessment will be extracted from the NorPD. The day of the diagnostic assessment conducted at the outpatient clinics is defined as the index date. Medications taken regularly are typically prescribed for three-month periods, thus we assume that capturing dispensed medications during the twelve months prior to the index date provides a valid estimate of drug exposure over time. The total number of drugs and the prevalence of polypharmacy will be calculated.

Drugs that, according to the 2023 update of the Beers Criteria and STOPP criteria version 3 (STOPPv3), should be avoided in individuals with MCI and dementia will be defined as PIMcog. Accordingly, benzodiazepines (BZDs), benzodiazepine derivatives, benzodiazepine-related drugs, antipsychotics, and drugs with strong anticholinergic properties will be categorized as PIMcog. Antipsychotics listed as anticholinergic drugs in BeersCriteria will be classified as anticholinergics. The complete list of PIMcog has been developed through adaptation to the Norwegian drug market (Supplementary S1 Table).

### Analysis plan

#### Statistical power and sample size.

This study is descriptive and aims to characterize PIMcog exposure over time; therefore, no statistical power calculations have been conducted. However, we have certain assumptions regarding sample size. In a previous study, we identified a prevalence of PIMcog use of 16% in a selected cohort of 397 patients with MCI and dementia attending Norwegian outpatient clinics from 2009 to 2016 [42]. That cohort contained a relatively high proportion of individuals with young-onset dementia and frontotemporal dementia and had a median age of 71 years. As the median age in the present study cohort is expected to be higher, and the proportion of individuals with frontotemporal dementia lower, we therefore expect the prevalence of PIMcog use to exceed 16% [37]. By including about 23,000 community-dwelling patients recorded in NorCog during 2014–2024, we estimate that approximately 20% (n = 4600) individuals will have been exposed to PIMcog during the year preceding the index date, enabling robust descriptive analyses of a broad group of PIMcog users. The prevalence of specific PIMcog classes and individual PIMcog agents is more difficult to anticipate. Based on previous studies, we expect prevalence rates of 5–10% for each PIMcog class, with z-hypnotics among the most frequently used PIMcog, with an expected prevalence of 20–25% [42,43]. Consequently, we anticipate sample sizes of at least 1,000–2,000 users for the most implicated PIMcog.

#### Statistical methods.

In this part, we will provide the descriptive analysis and statistical methods that will be performed in the study related to the objectives:

*Objective 1:* For each participant, the number of medications used during the 12 months preceding the index date will be calculated. The prevalence of polypharmacy (defined as the concurrent use of five or more medications), any PIMcog use, individual PIMcog classes, and specific PIMcog will be reported as frequencies and percentages for each calendar year during the study period. Temporal trends in polypharmacy and PIMcog use will be examined using logistic regression models with calendar year as the main independent variable. Odds ratio (OR) with corresponding 95% confidence interval (CI) will be presented. Temporal patterns in medication use will be visualized using graphical displays (e.g., annual prevalence plots). Where available and comparable, age-adjusted prevalence rates in the general population will be extracted from NorPD and used as a contextual reference.

*Objective 2:* Demographic, social, and clinical characteristics of PIMcog users and non-users will be compared using variables extracted from NorCog (Table 1) and the Charlson Comorbidity Index (CCI). The distribution of continuous variables will be assessed using normal probability plots and histogram. Continuous variables will be summarized using mean and standard deviations (SDs), and categorical variables as frequencies and percentages. Outliers will be inspected using box plot and scatter plot. Group comparisons will be performed using Chi-square or Fisher’s exact test for categorical variables, and Student’s t-test or analysis of variance (ANOVA) for continuous variables, as appropriate. Adjustments for multiple comparisons will be considered when relevant.

Univariate and multivariate logistic regression will be conducted to identify factors associated with PIMcog use. Potential explanatory variables will include the total number of drugs, CCI and the variables listed in Table 1. Model assumptions and fit will be assessed using appropriate diagnostic procedures.

For the multivariate models, correlations between explanatory variables will be examined, and the Variance Inflation Factor will be calculated to assess multicollinearity before variables are included in the models. As the CCI and total number of drugs are likely to be correlated, we will consider excluding one of the variables or combining highly correlated variables into composite measures, for example using principal component analysis.

Model fit will be evaluated using the Hosmer–Lemeshow test and other relevant diagnostic measures, as appropriate.

#### Inference criteria.

Statistical significance will be defined as a two-sided p-value < 0.05. Odds ratio with 95% CIs will be reported for regression analyses.

#### Software.

Statistical analyses will be performed using appropriate software, including STATA (StataCorp, College Station, TX, USA), IBM SPSS Statistics (IBM Corp, Armonk, NY, USA), or RStudio (Posit Software, Boston, MA, USA)

#### Missing data.

The extent and patterns of missing data will be examined. Sensitivity analyses will be performed to assess the potential impact of missing data. Where appropriate, missing values may be handled using multiple imputation methods. Variables that were not collected throughout the entire study period will not be considered missing and will not be imputed for periods in which they were not recorded.

### Data management and processing

Data extraction from NorCog has been approved by the NorCog Expert Council, which oversees and governs the use of clinical data for research purposes, as well as by the Data Protection Officer, ensuring compliance with applicable data protection regulations. Approval was granted on 02/12/2025, and the data have been made available for research purposes in alignment with this study protocol. However, the dataset has not yet been accessed and will not be released to the research team until the study formally commences.

All data will be stored in a dedicated project area on a secure server for sensitive information at Telemark Hospital Trust (O:Sensitive). Access to the project files will be restricted to members of the project group in accordance with approvals from the local data protection officer.

Data extracted from NorCog, including each participant’s personal identification number, will be linked with data from NorPD and NPR. Personal identification numbers will be replaced with study IDs. The code list linking study IDs and personal identification numbers will be stored separately from the research dataset in a dedicated secure file area (O:Sensitive), with access restricted to the PhD candidate and the project leader. Based on the code list and the date of diagnostic assessment (index date), we will obtain access to individual data on drug prescriptions from the NorPD and the comorbidity index from the NPR for all included participants. The participant’s personal ID number will be replaced by a unique pseudonym by Statistics Norway before disclosure from NorPD. Consequently, the file provided from NorPD includes each participant’s study ID number and a corresponding pseudonym together with information on the patient’s comorbidity and drugs dispensed during twelve months preceding the index date. The linked dataset will then be combined with variables extracted from NorCog.

All files sent and received will be encrypted with AES-256-bit. The password will be sent on a separate channel from the data file. The data will be processed and analyzed using statistical software such as SPSS, STATA and R as appropriate.

## Publications and timeline

The study will be published in a two-step Registered Report format. Data extraction from the registries and analysis will not commence until the present protocol has been peer-reviewed and accepted for publication (Stage 1).

In Stage 2, the full research article reporting the study findings will undergo a second round of peer review to ensure that the study has been conducted in accordance with the approved protocol.

Both stages will be submitted to and reviewed by the same journal. This two-step publication process increases transparency and reduces the risk of publication bias.

## Ethical considerations

In this study, we will use data from the national health registries NorCog, the NorPD and the NPR. Participation in NorCog is based on written informed consent in accordance with applicable regulations, including permission to link NorCog data with NorPD and NPR for research purposes.

Obtaining written informed consent from patients with cognitive impairment raises ethical challenges, particularly due to their potentially reduced capacity to fully understand the information provided and to give valid consent for participation in the registry. To address this, all patients are provided with both written and oral information. Assessment of each patient’s capacity to provide informed consent is performed by a specialist as part of the clinical evaluation. Participants are informed that they may withdraw their consent at any time without consequences.

Until January 2022, only patients with the capacity to provide informed consent were included. Since January 2022, patients who lack such capacity may be included based on written proxy consent. These procedures are intended to ensure ethically appropriate inclusion while preserving the representativeness of the study population.

All data handling and processing will be carried out in accordance with the General Data Protection Regulation (GDPR) and relevant Norwegian legislation, including the Health Register Act. The Data Protection Officer at Oslo University Hospital has approved the extraction of data from NorCog in accordance with the study protocol. The Data Protection Officer at Telemark Hospital Trust has approved the data processing prior to data collection and analysis (12/10/2025). NorPD is governed by national regulations of 10/17/2003, relating to the collection and processing of health data in NorPD. NorPD provides pseudonymized data files for research purposes, as regulated by the Norwegian law for health registers [44].

The study was approved by the Regional Committees for Medical and Health Research Ethics (reference number: 936497, approved 09/24/2025).

## Discussion

This protocol describes a nationwide registry-based study that will estimate the prevalence and temporal trends of polypharmacy and PIMcog among individuals assessed for cognitive symptoms and suspected of having dementia in Norwegian specialist outpatient clinics. Through linkage of NorCog with the NorPD and the NPR, the study will characterize medication use during the 12 months prior to the assessment while diagnoses, dementia severity, BPSD, and comorbidity will be assessed at the time of the NorCog registration.

In a previous study based on a small, selected sample of NorCog patients, we examined the medication exposure at and after diagnosis and focused on associations with subsequent changes in dementia severity and neuropsychiatric symptoms [42]. In contrast, the planned study will use nationwide registry data from all NorCog patients to investigate pre-diagnostic prescribing patterns at the population level. The study is designed to quantify prevalence, temporal trends over a 10-year period (2014–2024), regional variation, and clinical correlates of PIMcog use and polypharmacy, thereby addressing questions about how medications with cognitive adverse effects are used before patients reach specialist outpatient clinics.

Based on previous reports from other countries of polypharmacy and PIMcog prevalence among individuals with dementia, we hypothesize that the prevalence of polypharmacy has increased over time, and that PIMcog prescribing patterns differ between regions [45–47]. Moreover, we expect that PIMcog use is associated with higher disease burden, including more severe dementia, greater comorbidity, older age, higher overall medication use, and a higher prevalence of BPSD [12,42,48,49].

*Strengths:* The study has several methodological strengths. NorCog provides detailed, standardized clinical information from 46 outpatient clinics and covers a high proportion of individuals diagnosed with MCI and dementia in specialized healthcare in Norway, which supports generalizability within this setting. Linkage with NorPD enables near-complete capture of pharmacy-dispensed medications during the year before assessment, allowing robust estimates of overall polypharmacy and use of specific PIMcog. The NPR provides systematically collected diagnostic information from specialist healthcare, which will be used to derive comorbidity measures corresponding to the time of cognitive assessment. The combination of these three national registries, together with the long observation period, enables assessment of temporal trends in prescribing and identification of subgroups with particularly high medication burden and PIMcog exposure.

*Limitations:* This study has several limitations related to incomplete or missing information in the registries that should be acknowledged. First, NorCog includes patients referred to specialist outpatient clinics and may therefore not be fully representative of all individuals with cognitive impairment or dementia in Norway. The study is further restricted to community-dwelling individuals, as those receiving 24-hour residential care are excluded; this may limit the generalizability of the findings, particularly to frailer patients and individuals with advanced disease. Until the end of 2021, inclusion in NorCog required the capacity to provide informed consent, which likely led to underrepresentation of individuals with more severe cognitive impairment in the earlier years of the study period. In addition, neurological departments are not included in the registry, which may lead to underrepresentation of patients assessed in neurological settings and affect the overall representativeness of the study population. NorCog also lacks information on ethnicity, precluding analyses of ethnic differences in prescribing practices.

There are also limitations related to medication data. NorPD does not capture medications administered directly in institutions and hospitals, nor over-the-counter drugs, which may lead to underestimation of total medication exposure for some patients.

Finally, as an observational, registry-based study, the analyses depend on the accuracy and completeness of routinely collected clinical data. Although diagnoses in NorCog are established following comprehensive assessments in specialist outpatient clinics using recognized diagnostic criteria, some degree of diagnostic misclassification cannot be ruled out, particularly for MCI, a heterogeneous condition with inherent diagnostic uncertainty.

## Conclusion

This study is expected to provide important population-level data on pre-diagnostic medication use in individuals with MCI and dementia, which may inform clinical guidelines, support systematic medication reviews in diagnostic pathways, and help identify priorities for future interventional and longitudinal outcome studies.

## Supporting information

S1 Table List of PIMcog based on Beers 2023, STOPP v3 and adopted to the Norwegian drug market 2025. (DOCX)

## Abbreviations

BPSD: Behavioral and psychological symptoms of dementia
CCI: Charlson Comorbidity Index
CSDD: Cornell Scale for Depression in Dementia
CFS: Clinical Frailty Scale
MCI: Mild cognitive impairment
NPI-Q: Neuropsychiatric Inventory Questionnaire
NorCog: Norwegian Registry of Persons Assessed for Cognitive Symptoms
NorPD: Norwegian Prescribed Drug Registry
NPR: Norwegian Patient Registry
PIM: Potentially inappropriate medication
PIMcog: Potentially inappropriate medication for cognitive impairment
