# Supplementary material for: One year pre-diagnostic prevalence and associated factors of polypharmacy and potentially inappropriate medication use in community-dwelling adults with mild cognitive impairment or dementia in Norway (2014–2024): A registered report protocol
Source: PLoS One. 2026 Aug 18;21(8):e0356275. doi: 10.1371/journal.pone.0356275 (PMC13485053; doi:10.1371/journal.pone.0356275)
Supplement: Supplementary file 1 [file pone.0356275.s001.docx]

**S1 Table: List of PIMcog based on Beers criteria updated 2023, STOPP version 3 and adapted to the Norwegian drug market 2025**

| Drug substance | ATC code | Drug substance | ATC code | Drug substance | ATC code |
| --- | --- | --- | --- | --- | --- |
| Anticholinergics |  | **Benzodiazepines (BZD)/**  **BZD derivates and**  **BZD-related substances** |  | **Antipsychotics** |  |
| Scopolamine | A04AD01 | Diazepam | N05BA01 | Haloperidol | N05AD01 |
| Hyoscyamine | A03BA03 | Oxazepam | N05BA04 | Droperidol | N05AD08 |
| Oxybutynin | G04BD04 | Triazolam | N05CD05 | Sertindole | N05AE03 |
| Tolterodine | G04BD07 | Lorazepam | N05BA06 | Ziprasidone | N05AE04 |
| Solifenacin | G04BD08 | Alprazolam | N05BA12 | Lurasidone | N05AE05 |
| Darifenacin | G04BD10 | Flunitrazepam | N05CD03 | Quetiapine | N05AH04 |
| Fesoterodine | G04BD11 | Clonazepam | N03AE01 | Amisulpride | N05AL05 |
| *Biperiden | N04AA02 | Clobazam | N05BA09 | Risperidone | N05AX08 |
| Perphenazine | N05AB03 | Carbamazepin | N03AF01 | Aripiprazole | N05AX12 |
| Prochlorperazine | N05AB04 | Nitrazepam | N05CD02 | Paliperidone | N05AX13 |
| Chlorpromazine | N05AA01 | Midazolam | N05CD08 | Cariprazine | N05AX15 |
| *Chlorprothixene | N05AF03 | Zopiclone | N05CF01 | Brexpiprazole | N05AX16 |
| Clozapine | N05AH02 | Zolpidem | N05CF02 |  |  |
| Flupentixol | N05AF01 |  |  |  |  |
| Zuclopenthixol | N05AF05 |  |  |  |  |
| Olanzapine | N05AH03 |  |  |  |  |
| Hydroxyzine | N05BB01 |  |  |  |  |
| Levomepromazine | N05AA02 |  |  |  |  |
| Paroxetine | N06AB05 |  |  |  |  |
| Imipramine | N06AA02 |  |  |  |  |
| Clomipramine | N06AA04 |  |  |  |  |
| *Trimipramine | N06AA06 |  |  |  |  |
| Amitriptyline | N06AA09 |  |  |  |  |
| Nortriptyline | N06AA10 |  |  |  |  |
| Doxepin | N06AA12 |  |  |  |  |
| Alimemazine | R06AD01 |  |  |  |  |
| *Promethazine | R06AD02 |  |  |  |  |
| *Cyclizine | R06AE03 |  |  |  |  |
| Meclizine | R06AE05 |  |  |  |  |
| Doxylamine | R06AA09 |  |  |  |  |
| Doxylamine combinations | R06AA59 |  |  |  |  |
| Chlorpheniramine | R06AB02 |  |  |  |  |
| *Dexchlorpheniramine | R06AA61 |  |  |  |  |
|  |  |  |  |  |  |

Antipsychotics with moderate to strong anticholinergic effects are classified as anticholinergics. Anticholinergic drugs listed in Table 7 of the 2023 AGS Beers Criteria that are not marketed in Norway, or that are available only as parenteral formulations administrated in hospital settings, (17 drugs) were excluded.

*Drugs marketed in Norway that were added to our anticholinergic list were included based on strong anticholinergic effects reported in their Summaries of Product Characteristics and on the list of anticholinergic drugs published by the national network of medicines information and pharmacovigilance centers (RELIS) [1]

1.	Reiter L, Stenberg-Nilsen H, Okland HG. Use of anticholinergic drugs in older patients. Tidsskr Nor Laegeforen. 2021;141(6). doi: 10.4045/tidsskr.20.0775. PubMed PMID: 33876622.
